# Reporting environmental contamination results to healthcare workers could play a crucial role in decreasing the risk of occupational exposure to antineoplastic drugs

**DOI:** 10.3389/fpubh.2022.989977

**Published:** 2022-08-25

**Authors:** Alexandre Acramel, Sandy Blondeel-Gomes, Carla Matta, Subramanian Narayani, Olivier Madar, Romain Desmaris, Laurence Escalup, Julien Fouque

**Affiliations:** ^1^Département de Pharmacie, Institut Curie, PSL Research University, Paris, France; ^2^Université Paris Cité, CiTCoM, UMR8038 CNRS, U1268 Inserm, Paris, France; ^3^Département de Radiopharmacologie, Institut Curie, PSL Research University, Paris, France; ^4^Département d'Oncologie Médicale, Institut Curie, Saint-Cloud, France; ^5^Département d'Oncologie Médicale, Institut Curie, Paris, France; ^6^Département de Radiopharmacologie, Institut Curie, Saint-Cloud, France

**Keywords:** antineoplastic drugs, occupational exposure, environmental monitoring, wipe samples, hospital, healthcare worker, communication

## Introduction

Antineoplastic drugs (ADs) are still the standard treatment of cancer by acting on dividing cells to inhibit the uncontrolled reproduction of cancer cells but also on healthy cells by a non-targeted action. As a consequence, the majority of those medications are regarded as being hazardous to reproduction, carcinogenic, or mutagenic (CMR). In the past, nurses would prepare anticancer medications on the bench top without taking any special safety measures, which had negative effects on the workers who were exposed. There have been reported incidents of rashes, allergies, infertility, miscarriage, birth abnormalities, leukemia, and other malignancies (1). These hazardous drugs are also referenced in the monographs of the International Agency for Research on Cancer (IARC) according to a classification considering the risk of carcinogenicity for humans (2). Several methods have been used to assess occupational exposure directly in biological fluids or indirectly by searching for traces of ADs in the environment.

The implications of long-term exposure to ADs residues in hospitals are still unknown, despite the fact that this risk is now well-documented. The exposure is primarily caused by skin contact with contaminated surfaces. Subsequent biological (3) and toxicological (4) research also confirm that healthcare practitioners continue to be exposed to residual levels of contaminants. Hence, it's critical to manage and reduce the risk of exposure for healthcare professionals.

The European Union emphasized the significance of protecting workers who are exposed to carcinogens or mutagens as a result of the preparation, management, or disposal of hazardous drugs and all work involving exposure to carcinogens or mutagens in light of the fact that 1,5 million healthcare workers in Europe are exposed to ADs [DIRECTIVE (EU) 2019/130].

In western countries, injectable chemotherapy preparations are mainly centralized in hospital pharmacies. It has led to the implementation of additional protective measures throughout the chemotherapy process and setting of environmental monitoring (5) or healthcare workers biological fluids monitoring (3). Nevertheless, a lack of adherence to safety protecting measures and cleaning procedures (6–9), and poor knowledge of contamination risk (10–12) still described in healthcare population.

As we are convinced that the lack of communication conducts to a slackening of daily basis vigilance, we are focusing here on establishing proper feedback and discussions with healthcare workers regarding environmental monitoring campaigns.

## State of art

### Environmental monitoring: A useful tool since the 90's

Analysis of biological fluids are more informative about the contamination of healthcare workers than environmental monitoring but much more complex to conduct. Environmental monitoring by surface wipe sampling is the most commonly used method to evaluate the contamination throughout the chemotherapy process. It's therefore based on the choice of drugs tracers and the development of an exact, precise and as sensitive as possible analytical method (13).

Manual handling or automatic manufacturing of preparations, infusion of treatment, patients care waste management, and cleaning procedures are all steps during which the risk of contamination is present. Healthcare workers might be exposed when aerosols, leaking or spillage are generated, or when they come in contact with contaminated surfaces during the manufacturing of the preparations, infusion procedure disposal of waste, or cleaning (armchair, toilets, floor or bedding) (14–22).

Regular monitoring of environmental contamination has been carried out for several years in German (23), Italian (24), Czechoslovakian (19), Canadian (20) or American hospitals (21). These monitoring have shown that the risk of healthcare exposure is not systematically related to the level of environmental contamination or to the activity of the chemotherapy process but more to the practices and awareness of healthcare workers. However, these monitoring are useful to evaluate the efficacy of protective equipment, cleaning procedures (25–27), medical devices used for preparation or infusion [for example, Closed System Drug-Transfer Device (CSTDs) (28)], etc.

In some countries environmental monitoring are mandatory and some threshold values have been proposed to graduate the level of contamination and particularly for cyclophosphamide (23, 29–31). Considering the diversity of Ads used throughout the same facility, a multi-component analyzes is advisable. Analytical method must be representative of the activity and take into account the physio-chemical properties of the different ADs used. Several but reasonable number of tracers (5–10 tracers) should be considered but trying to analyze all the ADs of the chemotherapy process could complicate the interpretation. Liquid chromatography in tandem with mass spectrometry is an adequate method for environmental monitoring (13).

### Risk of occupational exposure: healthcare worker's view

Fazel et al. described in a recent paper the “barriers and facilitators for the safe handling” of ADs (10). Although there are recommendations on safe-handling of ADs, evidence suggests that compliance is usually very low. The most common barriers and facilitators identified in this review are, respectively, “poor training” and “adequate safety training.” These authors also emphasize the importance of “creating work environments where safety is a priority for the safe handling” of ADs.

In another paper, Boiano et al. described examples of activities which increase exposure risk reported by workers: “failure to wear appropriate nonabsorbent gown”; “intravenous tubing primed with antineoplastic drug”; “contaminated clothing taken home”; “spill or leak of antineoplastic drug during administration”; “failure to wear chemotherapy gloves”; and “lack of hazard awareness training” (8). In this study, respondents believed that dermal exposure to ADs was minimal and therefore did not wear the required PPE during administration. However, it has been demonstrated that skin contact during handling and administration is possible without precautionary work practices and use of personal protective equipment (PPE). Nowadays, dermal exposure resulting from skin contact with contaminated environmental surface is the main source of contamination. Similarly, despite the fact that safe handling recommendations have long been available, respondents did not always adhere to the advised procedures, highlighting the significance of training and education for both employers and employees. Curiously, the majority of respondents stated that they had received instruction on how to handle antineoplastic medications safely. The risk of exposure perceived by the workers is therefore an important factor in adherence to these safe handling recommendations. Thus, the authors suggest that “employers may be unaware of the adverse health risks,” but also that “better communication is needed to ensure that employers and workers are fully aware of the hazards and precautionary measures” to decrease exposures to ADs.

### Experience at Institut Curie: The CurieCONTA project

#### Annual environmental monitoring

We described in a recent paper a comparative study of environmental contamination by cyclophosphamide on the two hospital sites of Institut Curie (22). Not surprisingly, this work has shown that our preparation and administration areas are contaminated in very specific locations with cyclophosphamide and we know that other toxic drugs could be detected. The observations conducted in this study, allowed us to assess procedure compliance and identify potential determinants of environmental contamination.

**Figure 1:**
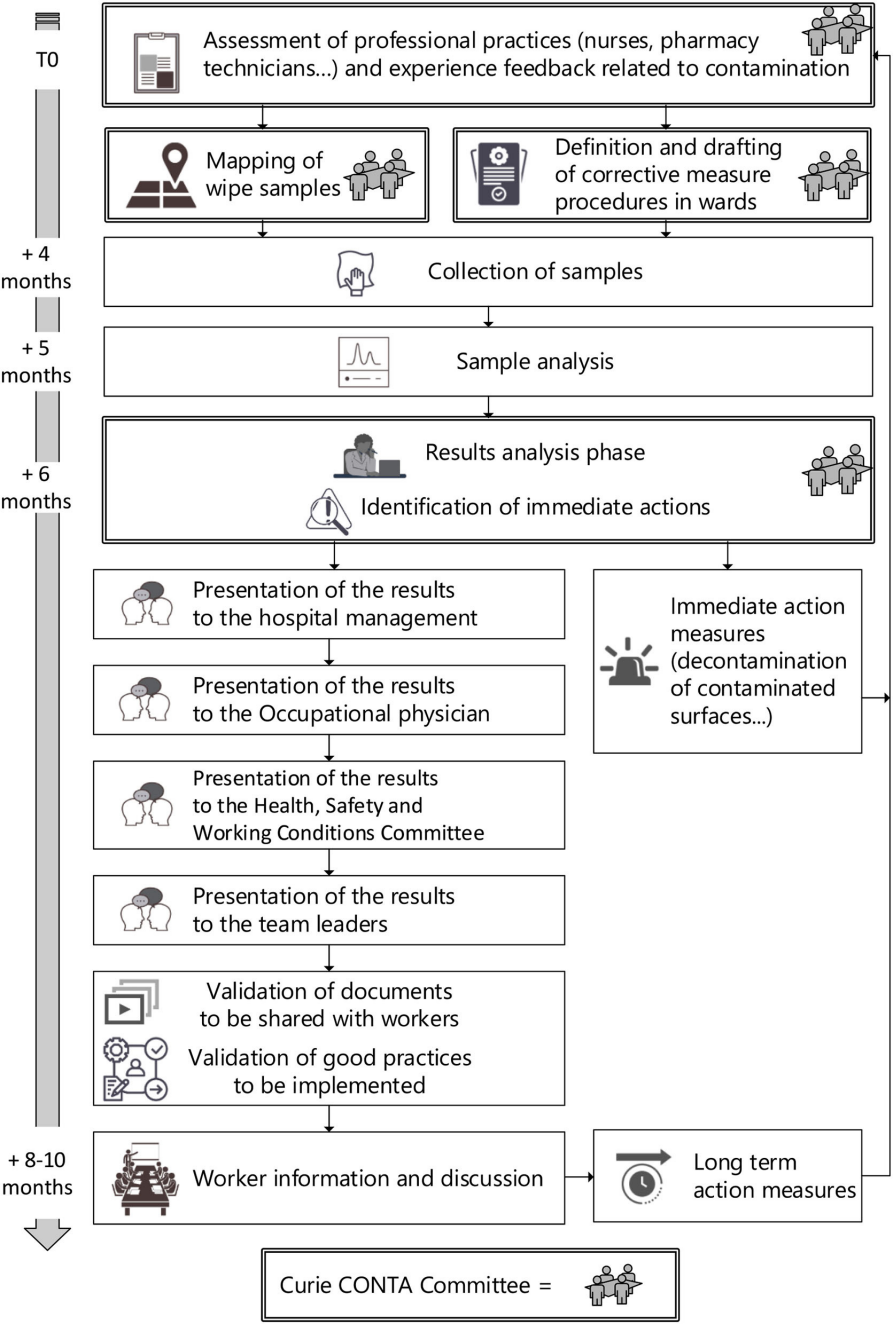
Annual environmental monitoring procedure and communication strategy at Institut Curie.

Recently, the French Agency for Food, Environmental and Occupational Health & Safety (Anses) published a report classifying work involving exposure to cytotoxic substances as carcinogenic processes. There is no obligation to make periodic environmental monitoring in France. However, identifying a few representative points of contamination and follow their evolution over times is a pertinent approach of quality improvement and risk management. It seemed essential to us to set up an environmental monitoring procedure in order to periodically check the state of contamination, to assess preventive measures, process changes or decontamination procedures. This also helps educational purposes, specifically to re-sensitize the healthcare workers who trivialize this risk as part of their daily practice. Considering the data available, an annual surface wipe sampling procedure was validated to assess the impact of the corrective measures. This annual surface wipe sampling procedure also include an assessment of professional practices and experience feedback related to contamination. This project, named “Curie CONTA,” is coordinated by a multidisciplinary working group (i.e., the Curie CONTA Committee) composed by pharmacists, pharmacologists, Occupational physicians, Health managers and a Hygiene Health Environment manager. The general procedure of this environmental monitoring is described in Figure 1.

#### Communication strategy

To our knowledge, the communication of results to healthcare workers is poorly detailed in the literature. That is why we proposed an example of annual communication strategy usually carried out in our hospital since 2018 (Figure 1). In the first place, the Curie CONTA Committee meets to discuss about the evolution of practices and feedback on contamination incidents, to define the mapping of the samples and the corrective measures to be evaluated during the environmental monitoring campaign. The collection of samples is planned with enough time to implement the corrective measures. After the analysis of the surface wipe samples, the results are discussed by the Curie CONTA Committee and if needed, immediate actions are validated. Then the results are presented to the management of the hospital, to the Occupational physician and to the Health, Safety and Working Conditions Committee. Finally, the results are presented to the healthcare workers including cleaning staff. These presentations are adapted for the different audience and validated by the Curie CONTA Committee This communication not only presents the evolution of the environmental contamination but also provides recommendations for controlling this risk of occupational exposure (i.e., long term action measures). Details of this annual environmental monitoring procedure and communication strategy is described in Figure 1.

We are convinced that this descending/ascending communication to healthcare workers is essential. It need to include every worker in order to answer questions, sensitize them to the risk of exposure, and encourage them to follow the defined recommendations. The feedback from the healthcare workers during these presentations are very positive. However, we should assess our approach, for example by using a questionnaire. In our experience, reporting these results of surface contamination measurements is an essential educational tool that raises awareness and helps healthcare professionals to decrease the risk of occupational exposure.

## Discussion

Even if ADs are defined as hazardous drugs, they are still extensively used in hospitals because of the continuous increasing number of cancers. Despite established guidelines, studies indicate poor compliance with current best practices, placing healthcare workers and their family at risk of exposure. The misuse of protective gloves and gowns suggest that there is a perception that exposures are inconsequential or so rare that they do not justify their use (8). The exposures observed through urine (32) or blood samples (33, 34) clearly reflect this lack of effectiveness or compliance with the preventive measures put in place.

Surface wipe sampling is now currently used as a standard method to determine workplace contamination in many countries. It is well-established that success of preventive and corrective measures occurred when surface contamination data are obtained but also properly communicated. The restitution of the results is therefore an important step for an awareness of the risk of exposure and a reminder of good practices. Improvements in prevention actions are therefore necessary and they must relate both to the information and training of workers and to the provision of suitable PPE and organizational measures allowing the control of contamination. An assessment of the impact and effectiveness of these preventive measures must therefore be carried out regularly. In the near future, we are waiting for a European harmonized definition of hazardous drugs. We are also waiting for new independent but comparable environmental monitoring studies from more and more hospitals. To help with this, those monitoring could be centralized by certified laboratories that have the expertise and the means to perform these analyses. Creating a European or International database and defining reference levels for hazardous drugs, specifically ADs would be an ambitious perspective but essential to meet the expectations of this issue. At last, we are also waiting for more ongoing training and education on this issue.

Finally, exposure monitoring and his management are essential. Our opinion is that reporting environmental contamination results to healthcare workers could play a crucial role in decreasing the risk of occupational exposure to hazardous drugs.

## Author contributions

All authors listed have made a substantial, direct, and intellectual contribution to the work and approved it for publication.

## Conflict of interest

The authors declare that the research was conducted in the absence of any commercial or financial relationships that could be construed as a potential conflict of interest.

## Publisher's note

All claims expressed in this article are solely those of the authors and do not necessarily represent those of their affiliated organizations, or those of the publisher, the editors and the reviewers. Any product that may be evaluated in this article, or claim that may be made by its manufacturer, is not guaranteed or endorsed by the publisher.
